# Effect of Cytokines on Osteoclast Formation and Bone Resorption during Mechanical Force Loading of the Periodontal Membrane

**DOI:** 10.1155/2014/617032

**Published:** 2014-01-19

**Authors:** Hideki Kitaura, Keisuke Kimura, Masahiko Ishida, Haruki Sugisawa, Haruka Kohara, Masako Yoshimatsu, Teruko Takano-Yamamoto

**Affiliations:** ^1^Division of Orthodontics and Dentofacial Orthopedics, Department of Translational Medicine, Tohoku University Graduate School of Dentistry, 4-1 Seiryo-machi, Aoba-ku, Sendai 980-8575, Japan; ^2^Department of Orthodontics and Dentofacial Orthopedics, Nagasaki University Graduate School of Biomedical Sciences, Nagasaki 852-8588, Japan

## Abstract

Mechanical force loading exerts important effects on the skeleton by controlling bone mass and strength. Several *in vivo* experimental models evaluating the effects of mechanical loading on bone metabolism have been reported. Orthodontic tooth movement is a useful model for understanding the mechanism of bone remodeling induced by mechanical loading. In a mouse model of orthodontic tooth movement, TNF-**α** was expressed and osteoclasts appeared on the compressed side of the periodontal ligament. In TNF-receptor-deficient mice, there was less tooth movement and osteoclast numbers were lower than in wild-type mice. These results suggest that osteoclast formation and bone resorption caused by loading forces on the periodontal ligament depend on TNF-**α**. Several cytokines are expressed in the periodontal ligament during orthodontic tooth movement. Studies have found that inflammatory cytokines such as IL-12 and IFN-**γ** strongly inhibit osteoclast formation and tooth movement. Blocking macrophage colony-stimulating factor by using anti-c-Fms antibody also inhibited osteoclast formation and tooth movement. In this review we describe and discuss the effect of cytokines in the periodontal ligament on osteoclast formation and bone resorption during mechanical force loading.

## 1. Osteoclast Differentiation

Osteoclasts, derived from hematopoietic stem cells, control bone resorption [1]. Two factors that influence the formation of mature osteoclasts have been identified. The first is receptor activator of NF-*κ*B ligand (RANKL) [2], also called osteoclast differentiation factor (ODF) [3], osteoprotegerin ligand (OPGL) [4], or TNF-related activation-induced cytokine (TRANCE) [5]. The second factor is macrophage colony-stimulating factor (M-CSF), which is essential for the proliferation and differentiation of osteoclast precursors [6]. Osteopetrotic (op/op) mice, which are deficient in M-CSF, show a lack of osteoclast development [7]. It has been reported that TNF-*α* mediates osteoclast formation *in vitro* [8–10] and *in vivo* [11, 12]. TNF-*α*-induced osteoclast recruitment is probably central to the pathogenesis of inflammatory disorders [13]. TNF-*α* is a known cause of rheumatoid arthritis [14], periodontal diseases [15], and postmenopausal osteoporosis [16]. TNF-*α* can induce biological reactions via two cell-surface receptors: TNF receptor type 1 (TNFR1) and TNF receptor type 2 (TNFR2). Each receptor mediates different intracellular signals. Analysis of TNFR1- and TNFR2-deficient mice revealed that TNFR1 induces osteoclast differentiation, while TNFR2 inhibits osteoclast differentiation [17]. The role of TNF-*α* signaling in osteoclastogenesis remains poorly understood, and further studies are needed to clarify the relationship between TNF-*α* and osteoclast differentiation.

## 2. Mechanical Loading

**Figure 1:**
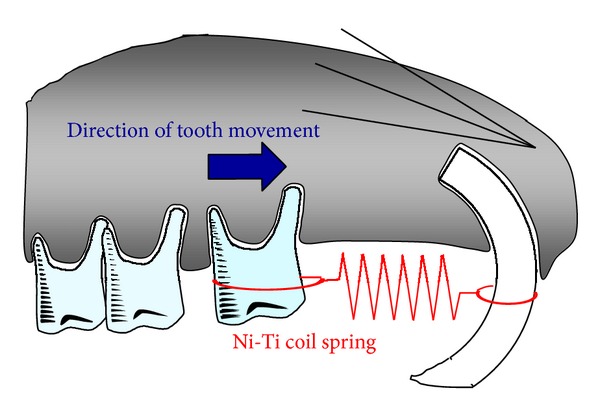
Schema of appliance for orthodontic tooth movement in mice. The orthodontic appliance is composed of a Ni-Ti coil spring. The appliance was inserted between the upper incisors and the upper left first molar and fixed with a 0.1 mm stainless wire around both teeth.

**Figure 2:**
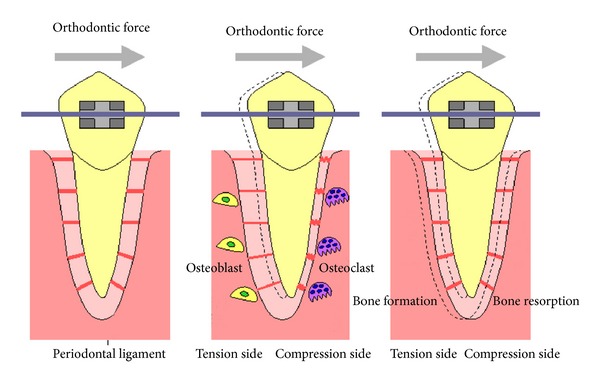
Schematic diagram of tooth movement. Applying orthodontic force to the tooth causes compression of the periodontal ligament. The compressed side of periodontal ligament is called the compression side and the side where the periodontal ligament is pulled is called the tension side. Osteoclasts appear on the compression side and osteoblasts on the tension side. The tooth moves as osteoclasts resorb bone while osteoblasts form bone.

Mechanical loading has important effects on skeletal bone mass and strength [18]. Several *in vivo* experiments have evaluated the effects of mechanical loading on bone metabolism, with mechanical loading caused by jumping [19, 20], treadmill running [21, 22], squatting [23], and swimming [24]. Orthodontic tooth movement is another useful *in vivo* model for elucidating the mechanism of mechanical loading-induced bone remodeling [25–28]. Orthodontic tooth movement has mainly been studied in rat and mouse models [29–37]. Recent advances in molecular biology techniques have provided opportunities for the use of gene-mutated mice, including those with mutations in genes that regulate bone metabolism. Mouse models of tooth movement can be advantageous in understanding the molecular mechanisms not only of tooth movement but also of mechanical loading-induced bone remodeling. In the mouse model, Ni-Ti coil springs are most suitable for exerting continuous orthodontic force [38, 39] (Figure 1). Orthodontic tooth movement is achieved by the process of alveolar bone resorption on the compression side and new bone formation on the tension side [40] (Figure 2). There is an association between osteoclasts and bone resorption on the compression side during orthodontic tooth movement [41]. In a mouse model, bone resorption and tartrate-resistant acid phosphatase- (TRAP-) positive multinuclear cells were recognized on the compression side.

## 3. TNF-*α*-Mediated Mechanical Loading-Induced Osteoclast Formation and Bone Resorption 

Mechanical forces affect tooth movement via the biological responses of cells in the periodontal ligament, the alveolar bone, and other paradental tissues [42]. Several cytokines and hormones are involved in this process. It has been reported that orthodontic tooth movement increases levels of TNF-*α* in the gingival sulcus in humans [43, 44]. It has been shown that TNF-*α* is expressed in rat periodontal tissue under pathological conditions resulting from excessive orthodontic force [45]. When a tooth movement system was applied to mice deficient in TNFR1 or TNFR2, less tooth movement was observed in TNFR2-deficient mice than in wild-type mice [38]. This result suggests that TNFR2 is important for orthodontic tooth movement. On the other hand, conflicting results were reported in a study of TNFR1- and TNFR2-deficient mice, which found increased osteoclast formation in TNFR1-deficient mice, with inhibited osteoclast formation in TNFR2-deficient mice [17]. Andrade et al. evaluated the effect of TNFR1 on osteoclast formation in orthodontic tooth movement. The number of osteoclasts in TNFR1-deficient mice was lower than in wild-type mice [46]. To further confirm the role of TNFRs, we performed tooth movement experiments using mice with mutations in both TNFR1 and TNFR2. We found a significant decrease in tooth movement in the double mutated mice [39]. These results suggest that TNF-*α* affects orthodontic tooth movement. However, the relationship between orthodontic movement and TNF-*α* is not fully understood.

## 4. Effect of Cytokines on Mechanical Loading-Induced Osteoclast Formation and Bone Resorption

Cytokines in the gingival area during orthodontic tooth treatment provide information about local cellular metabolism, reflecting the status of periodontal health and bone remodeling. Many investigators have found cytokine expression in the gingival area during orthodontic tooth movement. The course of osteoclast formation can be controlled by cytokines. Interleukin- (IL-) 6 [47], IL-17 [48], and transforming growth factor-*β* [49] induce osteoclast formation and increase bone resorption by osteoclasts. Conversely, IL-4 [50, 51], IL-10 [52], IL-12 [53–56], IL-13 [57], IL-18 [58–60], and IFN-*γ* [49, 61] inhibit osteoclast formation and several osteoclast functions. IL-4 [50, 51], IL-12 [55, 56], IL-18 [59, 60], and IFN-*γ* [61] inhibit TNF-*α*-induced osteoclast formation *in vitro* and *in vivo*. It has been reported that the cytokines IL-1*β* [62], TNF-*α* [38, 43, 44], IL-6 [63–65], IL-8 [64, 65], RANKL [66], M-CSF [67], TGF-*β* [68], IL-2 [65], and IFN-*γ* [69] were locally increased during orthodontic tooth movement. These cytokines may affect osteoclast formation during orthodontic tooth movement. We previously reported that TNF-*α* is expressed on the compression side of the tooth and plays an important role in mechanical tooth movement [38]. Therefore, we examined whether these cytokines inhibit mechanical tooth movement. We found that IFN-*γ* inhibited osteoclastogenesis during orthodontic tooth movement, suggesting that experimental tooth movement may cause TNF-*α*-induced osteoclastogenesis that is then inhibited by IFN-*γ* [69]. In another study using a rat model, IFN-*γ* inhibited osteoclast formation on the compression side during experimental tooth movement, as shown by immunohistochemical staining [70]. These results suggest that IFN-*γ* might control excessive osteoclastogenesis during orthodontic tooth movement. We previously demonstrated IL-12-induced apoptosis of osteoclast precursor cells during osteoclastogenesis [55, 56]. In these studies, we found that IL-12 inhibited TNF-*α*-mediated osteoclastogenesis by inducing apoptotic changes in osteoclast precursor cells through interactions between TNF-*α*-induced Fas and IL-12-induced FasL. We also investigated whether IL-12 inhibits mechanical tooth movement. IL-12 inhibited mechanical tooth movement through inhibition of osteoclastogenesis and bone resorption on the pressure side of teeth [71]. Many apoptotic cells were also recognized on the pressure side in IL-12-treated mice. Apoptosis may be caused by the interactions between TNF-*α*-induced Fas and IL-12-induced FasL in orthodontic tooth movement. Our results led us to conclude that IFN-*γ* and IL-12 induction inhibit osteoclastogenesis and tooth movement caused by mechanical force loading.

## 5. Effects of M-CSF on Mechanical Loading-Induced Osteoclast Formation and Bone Resorption

M-CSF is well known as an essential factor in osteoclast formation. It has been reported that administration of M-CSF receptor c-Fms antibody completely blocks osteoclastogenesis and bone erosion induced by TNF-*α* administration or inflammatory arthritis [12]. Orthodontic tooth movement is also mediated by TNF-*α*. Therefore, we hypothesized that anti-c-Fms antibody might block osteoclastogenesis and bone resorption at the compression side of a tooth undergoing orthodontic tooth movement. In our study, anti-c-Fms antibody injected daily into a local site for 12 days during mechanical loading significantly inhibited orthodontic tooth movement and markedly reduced the number of osteoclasts *in vivo* [39]. Brooks et al. showed that injection of M-CSF accelerated orthodontic tooth movement and osteoclast formation [72]. These results suggest that control of M-CSF could regulate osteoclast formation and tooth movement in orthodontic treatments. The receptor tyrosine kinase inhibitor SU11248 prevents activation of the M-CSF receptor, inhibiting osteoclast formation and function *in vitro* and *in vivo* [73]. The tyrosine kinase inhibitor imatinib also inhibits the M-CSF receptor. These results suggest the possibility of drug treatment for bone destruction [74]. However, the therapeutic use of M-CSF must be approached with caution because significant complications have been encountered with other forms of anticytokine therapy [75]. Further studies are necessary to evaluate the therapeutic use of M-CSF.

## 6. Root Resorption 

Root resorption is a possible complication of orthodontic treatment and is a serious problem for orthodontists. Several studies have suggested that excessive orthodontic force is a critical factor in root resorption [76, 77]. It has been reported that root resorption is associated with tooth morphology [78], tooth intrusion [79, 80], periodontal condition [81], and systemic factors such as genetics [82], the immune system [83, 84], and bone metabolism [85, 86]. In our mouse orthodontic tooth movement system, a Ni-Ti coil spring was inserted between the upper incisors and the upper first molar. Root resorption occurred in this model [87]. Root resorption results from the activity of odontoclasts, which play a role similar to that of osteoclasts in bone resorption. Like osteoclasts, odontoclasts are multinucleated giant TRAP-positive cells with ruffled borders [88]. Tsuchiya et al. reported that odontoclasts had fewer nuclei, smaller TRAP-positive area, and higher expression of MMP-9 than osteoclasts [89]. It remains unclear whether odontoclasts and osteoclasts can be considered functionally identical. We tested our hypothesis that IL-12 and anti-c-Fms antibody might inhibit odontoclastogenesis and root resorption during orthodontic tooth movement by injecting IL-12 locally adjacent to the first molar every other day during the experimental period. We found that IL-12 inhibited odontoclastogenesis and root resorption during orthodontic tooth movement [71]. Anti-c-Fms antibody also significantly inhibited odontoclastogenesis and root resorption during orthodontic tooth movement [87]. M-CSF and its receptor are potential therapeutic targets in mechanical stress-induced odontoclastogenesis, and injection of an anti-c-Fms antibody might be useful to prevent mechanical stress-induced root resorption during orthodontic tooth movement.

## 7. Conclusion

Many studies have reported the expression of various cytokines during mechanical loading of the periodontal ligament. Several studies using gene-mutated mice have shown that TNF-*α* plays a key role in mechanical force loading-induced osteoclast formation in the periodontal ligament. Therefore, it is important to study the relationship between TNF-*α*-induced osteoclast formation and cytokines expressed during mechanical loading. Further studies are needed to fully understand the effect of cytokines on mechanical loading-induced osteoclast formation.
